# Volumetric motion quantification by 3D tissue phase mapped CMR

**DOI:** 10.1186/1532-429X-14-74

**Published:** 2012-10-26

**Authors:** Anja Lutz, Jan Paul, Axel Bornstedt, G Ulrich Nienhaus, Patrick Etyngier, Peter Bernhardt, Wolfgang Rottbauer, Volker Rasche

**Affiliations:** 1 Department of Internal Medicine II, University Hospital of Ulm, Ulm, Germany; 2 Department of Diagnostic and Interventional Radiology, University Dusseldorf, Dusseldorf, Germany; 3 Institute of Applied Physics and Center for Functional Nanostructures (CFN), Karlsruhe Institute of Technology (KIT), Karlsruhe, Germany; 4 Medisys Research Lab, Philips Healthcare, Suresnes, France

**Keywords:** Velocity encoding, 3D-TPM, Motion quantification parameters, Myocardial motion

## Abstract

**Background:**

The objective of this study was the quantification of myocardial motion from 3D tissue phase mapped (TPM) CMR. Recent work on myocardial motion quantification by TPM has been focussed on multi-slice 2D acquisitions thus excluding motion information from large regions of the left ventricle. Volumetric motion assessment appears an important next step towards the understanding of the volumetric myocardial motion and hence may further improve diagnosis and treatments in patients with myocardial motion abnormalities.

**Methods:**

Volumetric motion quantification of the complete left ventricle was performed in 12 healthy volunteers and two patients applying a black-blood 3D TPM sequence. The resulting motion field was analysed regarding motion pattern differences between apical and basal locations as well as for asynchronous motion pattern between different myocardial segments in one or more slices. Motion quantification included velocity, torsion, rotation angle and strain derived parameters.

**Results:**

All investigated motion quantification parameters could be calculated from the 3D-TPM data. Parameters quantifying hypokinetic or asynchronous motion demonstrated differences between motion impaired and healthy myocardium.

**Conclusions:**

3D-TPM enables the gapless volumetric quantification of motion abnormalities of the left ventricle, which can be applied in future application as additional information to provide a more detailed analysis of the left ventricular function.

## Background

Cardiac motion quantification of the whole left ventricle with tissue phase mapping (TPM) appears important for improved understanding of the myocardial motion pattern as well as for improving diagnosis and therapy in many cardiac diseases like asynchrony or left bundle brunch block (LBBB). Due to the long acquisition times in TPM CMR, parameters quantifying myocardial motion are mainly derived from multi-slice TPM data or even from multi-slice tagging CMR. However, the availability of a full 3D motion field over the entire left ventricle appears attractive for a more detailed understanding of the motion pattern abnormalities in patients. Especially patients referred for cardiac resynchronization therapy might benefit from a more accurate motion analysis. Up to now, criteria for patients undergoing cardiac resynchronization therapy are New York Heart Association (NYHA) class 3 or 4, LVEF ≤ 35% and QRS duration > 120ms [1]. These criteria are not sufficient to predict the response to CRT, since a substantial percentage of patients (about 30%) does not benefit from the biventricular pacemaker therapy [1]. A more detailed analysis of the myocardial motion pattern appears as valuable further input for improving the prediction of response to CRT.

Different imaging techniques accelerating data acquisitions have been introduced including local imaging techniques reducing the field of view to a specific area [2-4], techniques using temporal correlations like view sharing [5-8], techniques using correlations in k-space or image space like generalized autocalibrating partial parallel acquisitions (GRAPPA) [9] and sensitivity encoding (SENSE) [10] , as well as techniques using both, correlations in k-space and time, like k-t BLAST, k-t SENSE, k-t GRAPPA and k-t PCA/SENSE [11-14].

View sharing, SENSE and k-t BLAST have been applied to TPM of the left ventricular myocardium [8,15,16]. Without losing substantial information of the motion pattern, view sharing enables a reduction of the overall TPM image acquisition time by 37.5% [8], k-t BLAST by 50% [15] and SENSE by 75% [16]. Thus SENSE acceleration appears as the most promising candidate for establishing a volumetric TPM data acquisition.

Quantitative parameters retrieved from multi-slice tagging and velocity encoded data have been introduced to assess the twisting motion of the heart as well as its asynchrony. Parameters were derived from velocity-time curves, torsion-time curves, rotation angle-time curves or strain-time curves. The goal of these parameters is to distinguish between different myocardial motion pattern and to enable automatic identification and quantification of motion abnormalities.

### Parameters derived from velocity-time curves

Parameters derived from velocity-time curves were introduced for tissue phase mapped imaging and include the standard deviation of the time to the systolic and diastolic peak [17] and the asynchrony correlation coefficient [18]. Reduced systolic and diastolic velocities have been reported in patients selected for cardiac resynchronization patients (CRT) and patients suffering from myocardial infarction [19,20].

The standard deviation of the time to systolic and diastolic peak (σ_TTP_) has been assessed and increased σ_TTP_ values have been found to be significantly increased in DCM patients [17].

The asynchrony correlation coefficient (ACC) quantifies the synchrony of the left ventricular contraction. High values indicate synchronous motion, whereas low values represent asynchronous motion. Compared to healthy volunteers, reduced ACCs have been found in regions with myocardial infarction [18].

### Parameter derived from torsion rate-time curves

The peak systolic and peak diastolic torsion rate has been introduced by Petersen et al. [21] as a system independent measure of the motion derived from the normalized differences between basal and apical circumferential velocity.

### Parameter derived from rotation angle-time curves

The base apex rotation correlation (BARC) quantifies the correlation between the apical and basal rotational motion. It is calculated from the apical and basal rotation angle-time curves and has been shown to provide a predictive value for the response to CRT [22].

### Parameters derived from strain-time curves

Parameters derived from stain-time curves have been introduced for tagged CMR data and include the temporal uniformity of strain [23-26], the standard deviation of onset time of different cardiac segments [27,28], the onset of shortening (OS) and peak of shortening (PS) delay vector [27,29], the regional variance of strain and regional variance of principle strain [23], the coefficient of variation [28,30] and the difference between septal and lateral strain at peak shortening of circumferential strain [28].

The temporal uniformity of strain (TUS) quantifies the synchrony of myocardial motion [23-26]. High circumferential TUS values have been reported for healthy volunteers, whereas TUS is reduced for CRT heart failure patients [26].

The standard deviation of the onset time T_onset_ of the contraction of different segments of the heart has been calculated in [28] as a quantitative measure of synchrony of motion. Increased differences of T_onset_ in different myocardial segments have been reported in patients referred for biventricular pacing [27].

The onset and peak time of strain T_onset_ and T_peak_ have been used to calculate the OS delay vector and PS delay vector describing the delay of T_onset_ and T_peak_ between different myocardial regions [27,29]. Different delay vectors have been found between patients screened for CRT and healthy volunteers [27].

Decreased regional variance of strain (RVS) values have been reported in dogs with cardiac failure and left bundle conduction delay after biventricular pacing compared to RVS after left-ventricular pacing and after right-atrial asynchronous pacing [23].

The regional variance vector of principle strain (RVVPS) is introduced in Helm et al. [23] and is expected to be low for synchronous motion. It has been shown in dogs with cardiac failure and left bundle conduction delay that RVVPS is smaller after biventricular pacing compared to RVVPS after left-ventricular pacing or right-atrial asynchronous pacing [23].

The coefficient of variation (CV) describes the standard deviation of strain divided by the mean value at a given time point [28,30]. Increased values of CV have been reported in patients with DCM and LBBB compared to healthy volunteers [30].

The difference between the septal and lateral strain at peak shortening of circumferential strain DiffSLpeakCS has been introduced in [28]. For ischemic patients DiffSLpeakCS is increased compared to nonischemic patients [28].

The objective of this study is to investigate the feasibility of application of volumetric tissue phase mapping for quantification of myocardial motion parameters based on velocity, displacement and strain data. It is shown that quantitative motion parameters originally derived from either TPM or tagging data can be derived from the isotropic velocity field.

## Methods

### Volunteers and patients

12 adult volunteers (7 males, 5 females, age 26 ± 7 years) without known cardiac disease and 2 patients (DCM patient: male, 46 years, DCM and asynchrony, LBBB patient: male, 29 years, LBBB and asynchrony) were enrolled in this study. The study protocol was approved by the local ethics committee. Written informed consent was obtained from all volunteers and patients prior to the MR examination.

### Data acquisition

Data acquisition was performed on a 3T whole body MR scanner (Achieva 3.0T, Philips, Best, The Netherlands) with a 32 channel phased array cardiac coil.

A vector ECG was applied for cardiac triggering. A volumetric 3D black blood velocity encoded respiratory navigated segmented gradient echo sequence (3D-TPM) was applied for coverage of the whole left ventricle [16]. The acquisition protocol is listed in Table 1. Please note that the field-of-view has to be enlarged in patients to account for the enlarged left ventricle dimensions.

**Table 1:** Acquisition parameters for the volunteer group as well as for both patients

**Parameter**	**Volunteers**	**DCM patient**	**LBBB patient**
FOV (M × P × S) [mm^3^]	380 × 380 × 63	320 × 320 × 90	330 × 330 × 81
Acq. Matrix (M × P)	128 × 124	112 × 100	112 × 100
Flip angle [°]	15	15	15
T_R_[ms]/T_E_[ms]	7.1 / 4.9	7.1 / 4.6	7.1 / 4.6
k-lines per segment	3	3	3
Phase interval [ms]	37.3	33.3	33.3
n_ph_	25	25	25
n_sl_	21	30	27
Resolution [mm^3^]	3 × 3 × 3	3 × 3 × 3	3 × 3 × 3
VENC [cm/s]	20	30	30
Navigator duration [ms]	15.5	15.5	15.5
Navigator feedback time [ms]	5	5	5
Navigator acceptance window [mm]	5	5	5
SENSE factor	4	4	4
Sub-volumes	3	3	3
Nominal scan duration [min:s] (assuming a navigator efficiency of 100%)	15:30	18:06	16:18
Actual acquisition duration [min:s]	33.55 ± 6:56	44:08	39:45
Navigator efficiency [%]	47.25 ± 8.59	41	41

Images were orientated in short axes geometry. The velocity encoding was performed in all three spatial directions in consecutive heart beats in order to increase the number of measurable cardiac phases [15,16,19].

To avoid flow artefacts in the phase contrast images and to ensure a good delineation between myocardium and blood, black blood contrast was performed by the application of two presaturation slabs next to the imaged volume. To ensure sufficient black blood contrast over the entire volume, the 3D volume covering the left ventricle was divided into three distinct sub-volumes covering the apical, equatorial and basal regions of the left ventricle. The three sub-volumes were acquired in one acquisition thus using the same navigator for all sub-volumes. The presaturation slabs were applied alternately to reduce the specific absorption rate [31].

### Data analysis

The 3D-TPM data were processed by in-house developed MATLAB programs (Matlab 2010b; Mathworks, Natick, Mass). The segmentation of the myocardial muscle was performed semi-automatically. After manual detection of the myocardium in one systolic and one diastolic cardiac phase, the propagation of the endo- and epicardial contours was performed automatically relying on active contour techniques and a shape model [32,33]. To avoid phase errors due to field inhomogeneities and eddy currents background phase error correction was applied using a linear fit to the phase of static tissue [34].

From the 3D-TPM data velocity-time curves (v-t curves), torsion rate-time curves (T-t curves), rotation angle-time curves (α-t curves), and strain-time curves (s-t curves) were extracted. V-t curves were calculated in longitudinal (towards the apex of the heart), radial (towards the center of the blood pool) and circumferential (in clock-wise direction) direction for each slice. Thereby the velocity was averaged over the whole myocardium or a segment of the myocardium of the respective imaged slice. Prior to the analysis and calculation of motion estimation parameters, the velocity data was interpolated over time by cubic splines [16].

Torsion-time curves were derived as described by Petersen et al. [21].

For the calculation of α-t and s-t curves, the velocities were converted to displacement data similar to the forward tracking in Pelc et al. [35]. The location x_t_ of a point x at time t was calculated by

$$x_{t}=\left(v\left(t-1\right)+v\left(t-2\right)+\cdots +v\left(0\right)\right)\Delta t+x_{0},$$

where x_0_ is the initial position of the point, v is the velocity and Δt is the distance between two investigated time points. Thereby, the velocity was averaged over the investigated segments before the calculation of the displacement data.

The rotation angle relative to the start position was calculated from the resulting positions over the cardiac cycle.

The calculated positions were additionally used to calculate s-t curves. Circumferential strain was calculated as length change of points on the myocardial centerline between subsequent time points, radial strain as length change between points lying on a normal vector to the myocardial centerline.

### Parameters derived from velocity-time curves

Investigated quantitative parameters based on v-t curves were the standard deviation of peak systolic and diastolic velocities [σ_TTP_], the asynchrony correlation coefficient [ACC], the global velocity ranges Δv and the temporal uniformity of velocity [TUV].

For the calculation of σ_TTP_, the myocardium was divided into n_seg_ = 6 segments in each slice and the peak systolic and diastolic velocities were determined for each segment. The standard deviation of the time to peak systolic velocities (*σ*_TTP_^sys^) and the standard deviation of the time to peak diastolic velocities (*σ*_TTP_^dias^ were calculated over all segments. This evaluation was performed for both longitudinal and radial v-t curves. The corresponding standard deviations were denoted as *σ*_TTP_^sys,1^,  *σ*_TTP_^dias,1^,  *σ*_TTP_^sys,r^*and σ*_TTP_^dias,r^.

For the calculation of the ACC the myocardium of each slice s was divided into n_seg_ = 24 segments. Let v(i,s,t) be the velocity of segment i at time step t in slice s and v(s, t) the averaged velocity of slice s at time step t. The mean values over time were given by $\bar{v}\left(i,s\right)$ and $\bar{v}\left(s\right)$. The asynchrony correlation coefficient for each segment i in the investigated slice s was defined by

$$\begin{array}{l} ACC\left(i,s\right)\\ \;=\frac{\sum_{t}^{n_{t}}\left(v\left(i,s,t\right)-\bar{v}\left(i,s\right)\right)\left(v\left(s,t\right)-\bar{v}\left(s\right)\right)}{\sqrt{\sum_{t}^{n_{t}}{\left(v\left(i,s,t\right)-\bar{v}\left(i,s\right)\right)}^{2}}\sqrt{\sum_{t}^{n_{t}}{\left(v\left(i,s,t\right)-\bar{v}\left(i,s\right)\right)}^{2}}}\\ \;\in \left[-1;1\right] \end{array}$$

where n_t_ is the number of time steps. The asynchrony correlation coefficient was determined for each segment in each slice. In this contribution the mean ACC ($\overset{―}{ACC}$), minimum ACC (ACC_min_) and maximum ACC (ACC_max_) were also calculated. The ACC was initially introduced for radial velocities [18]. In this study, the ACC is additionally calculated for longitudinal and circumferential velocities.

The velocity range for each slice was defined as *Δv* = *v*_*max*,*sys*_ − *v*_*min*,*dias*_. The global velocity range $\bar{\Delta v}$ was calculated by averaging the velocity range over all acquired slices. The global velocity range was determined for longitudinal ${\bar{\Delta v}}_{1}$ as well as for radial ${\bar{\Delta v}}_{r}$ v-t curves.

The temporal uniformity of velocity [TUV] was derived from the similar but strain-based parameter temporal uniformity of strain [TUS]. For the calculation, the myocardium of each slice was divided into n_seg_ = 24 segments. For each segment i of the investigated slice s and each time step t the velocity v(i, s, t) was calculated. For a given slice s and time step t the velocity was plotted against the myocardial segments i. Afterwards, a Fourier Transformation was performed. Assuming a similar velocity over all segments, only the zero-order Fourier term S_0_(t, s) would be unequal to zero, whereas asynchronous motion would result in a first-order Fourier term S_1_(t, s) unequal to zero. The TUV value for a specific slice s was calculated as

$$\begin{array}{l} TUV\left(s\right)=\sqrt{\frac{S_{0}}{S_{0}+S_{1}}}\;\text{with}\;S_{0}=\sum_{t=1}^{n_{t}}S_{0}\left(t,s\right)\;\text{and}\\ \;S_{1}=\sum_{t=1}^{n_{t}}S_{1}\left(t,s\right) \end{array}$$

TUV was averaged over all slices: $TUV=\sum_{s=1}^{n_{s}}TUV\left(s\right)$, where n_s_ is the number of acquired slices. The parameter TUV was calculated for all investigated velocity directions (TUV_l_, TUV_r_ and TUV_c_).

### Parameter derived from torsion rate-time curves

The peak systolic and peak diastolic torsion rate were determined from the torsion rate-time curves as described in Petersen et al. [21].

### Parameter derive from rotation angle-time curves

The base apex rotation correlation [BARC] was derived from α-t curves. This parameter was calculated as described in [22] with the difference, that in our study the correlation between the apical and basal rotation was measured up to the diastolic resting phase, whereas in [22] the analysis was stopped at the time of mitral valve opening.

### Parameters derived from strain angle-time curves

Investigated quantitative parameters based on s-t curves were the temporal uniformity of strain [TUS], the regional variance of strain [RVS], the regional variance vector of principle strain [RVVPS], the standard deviation of onset and peak time [SD(T_onset_) and SD(T_peak_)], the onset of shortening [OS] and peak of shortening [PS] delay vector, the coefficient of variation [CV] and the difference between septal and lateral peak circumferential strain [DiffSLPeakCS].

TUS was determined as described in [23-26]. The number of segments per slice was 24. TUS was calculated for both the circumferential and the radial strain [TUS_c_ = CURE and TUS_r_.

RVS was calculated for every time point and defined as the variance of strain over all segments and all slices as described previously [23]. The number of segments per slice used in this study was n_seg_ = 24. The maximum of RVS over time (RVS_max_) was calculated for the circumferential strain.

RVVPS was calculated for every time point as described in [23]. The number of segments per slice used in this study was n_seg_ = 24. The maximum of RVVPS over time (RVVPS_max_) was determined. RVVPS was calculated for the circumferential strain.

For the calculation of SD(T_onset_) and SD(T_peak_) the myocardium of each slice was divided into n_seg_ = 6 segments. The onset of circumferential shortening time and peak time were determined [27,29]. The standard deviation of T_onset_ and T_peak_ over all segments of all slices was calculated for the circumferential strain.

The OS and PS delay vectors were calculated as described in [27,29]. The components of the vectors were defined as differences of T_onset_ or T_peak_ between septal and lateral wall [SL], inferior and anterior wall [IA] and apical and basal wall [AB]. In our study, we used the most apical and most basal reconstructed slice for the calculation of the third component of the OS and PS delay vector. In case of the first and second component, the delay vectors were calculated for each slice and afterwards averaged over all slices. The OS and PS delay vectors were calculated for the circumferential strain.

CV was calculated as described in [30] and was defined as standard deviation of the strain in each segment multiplied by 100% divided by the mean value of strain at the time point of maximal contraction. The number of segments per slice used here was n_seg_ = 6. CV was calculated for the circumferential strain.

DiffSLPeakCS was determined as described in [28]. It is based on the differences between the peak septal and lateral circumferential strain for each segment and slice. The final parameters results as the mean value over all slices.

## Results

Table 1 provides the mean and standard deviations of the navigator efficiencies and the actual acquisition durations. The navigator efficiency of all volunteers and patients was between 30% and 60%, the actual acquisition duration was between 25 and 52 minutes. Despite the long acquisition times, image quality was sufficient for the analysis of motion analysis in all subjects and no limiting respiratory artefacts were observed.

**Figure 1:**
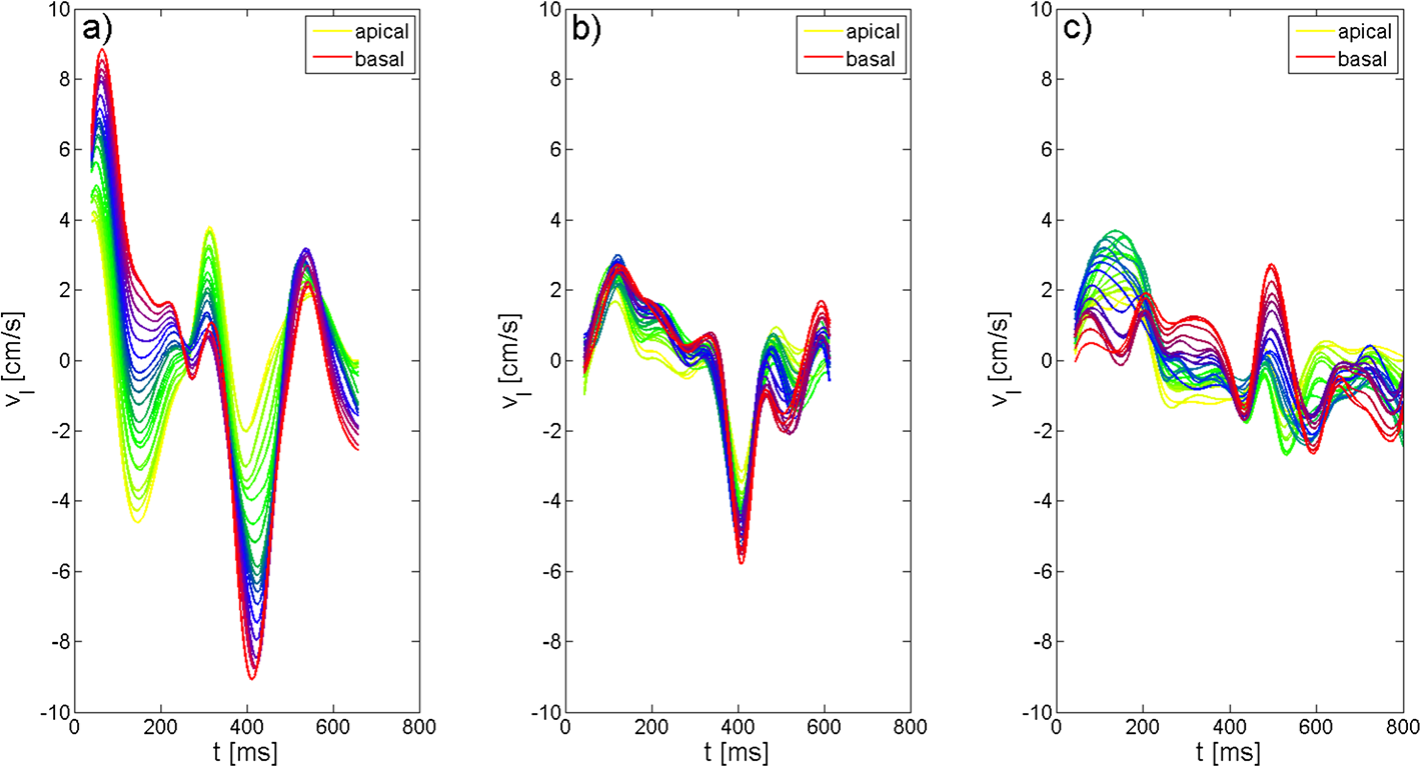
**Longitudinal velocity-time curves for a healthy volunteer and both patients obtained by 3D-TPM.** Longitudinal velocity-time curves are displayed for all investigated slices for a healthy volunteer (**a**), the DCM patient (**b**) and the LBBB patient. Huge differences between the longitudinal motion obtained in the volunteer and in the patients are observed.

Figure 1 displays the longitudinal motion exemplary for one volunteer (Figure 1.a), the patient with DCM and asynchrony (Figure 1.b) [DCM patient] and the patient with LBBB and asynchrony (Figure 1.c) [LBBB patient].

In general, the observed longitudinal, radial and circumferential motion patterns are similar for all investigated volunteers. Deviation from these motion patterns were clearly identified in our two patients.

In volunteers, the longitudinal motion starts with a global movement towards the apex of the heart. This motion is stronger for basal than for apical slices. At end-systole, the velocity is decreased to a plateau in the basal slices, whereas equatorial and apical slices are reaching small negative values. In diastole, all slices move back towards the basis of the heart. This motion is again more pronounced for basal than for apical slices. Subsequently a short motion in opposite direction occurs in all regions.

Major differences between the longitudinal motion pattern of the volunteers and the motion pattern of our two patients are the reduced systolic and diastolic velocities, the broadened systolic peak and the occurrence of the plateau in all locations. No further differences in the motion pattern can be observed in the DCM patient, whereas in the LBBB patient the longitudinal velocity performs a biphasic pattern with even positive velocities at mid-diastole.

**Figure 2:**
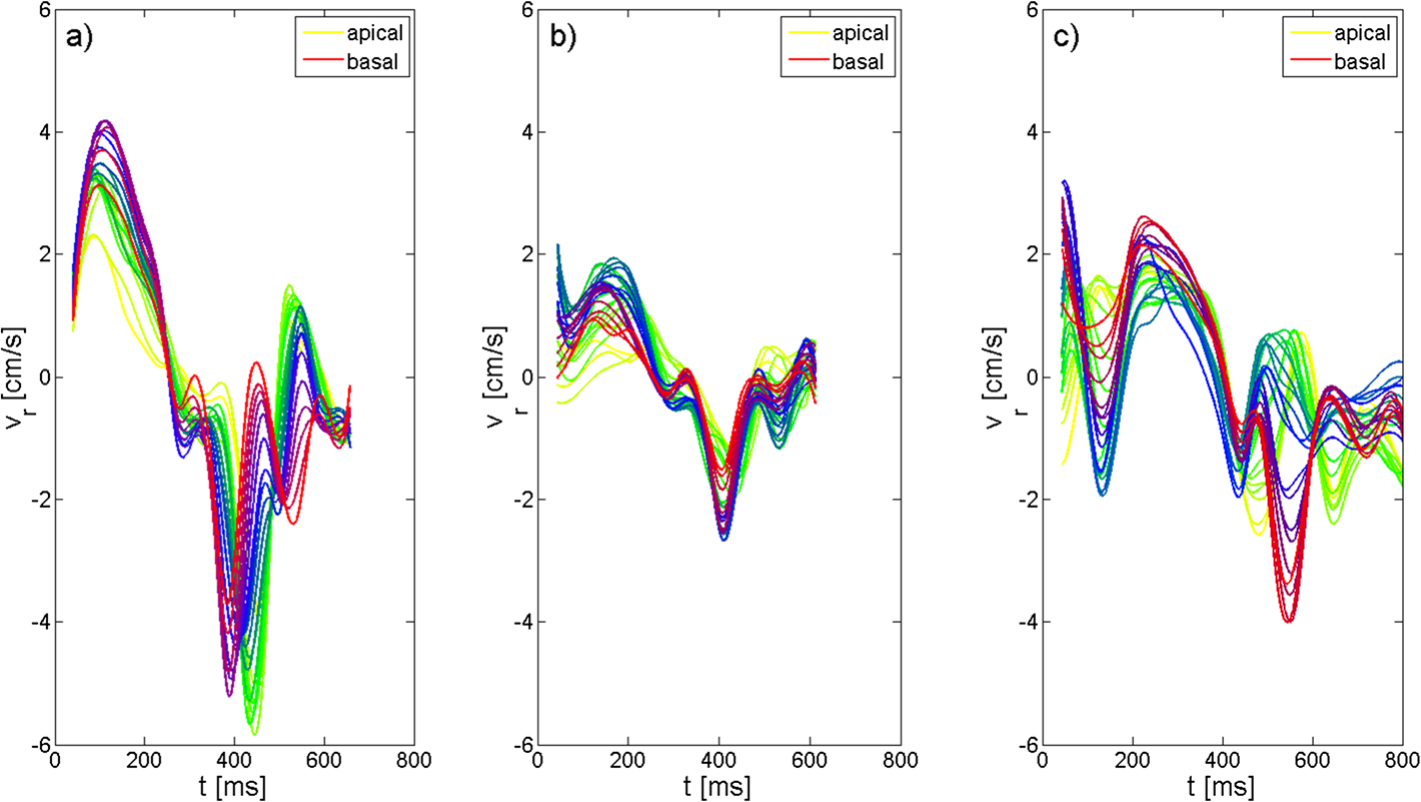
**Radial velocity-time curves for a healthy volunteer and both patients obtained by 3D-TPM.** Radial velocity-time curves are displayed for all investigated slices for a healthy volunteer (**a**), the DCM patient (**b**) and the LBBB patient. Huge differences between the radial motion obtained in the volunteer and in the patients are observed.

Figure 2 displays the radial motion exemplary for one volunteer (Figure 2.a), the DCM patient (Figure 2.b) and the LBBB patient (Figure 2.c).

In volunteers, during systole all slices move towards the center of the myocardium. Highest radial velocities are obtained in apical and equatorial slices. During diastole, all slices move back towards their original position. In apical slices, this occurs in a single distinct outward motion, whereas towards the basis of the heart two peaks located around the apical peak can be observed.

Major deviations between the radial motion pattern of the volunteers and the motion pattern of our two patients are reduced systolic and diastolic velocities. Whereas in the DCM patient the main effect is limited to the reduction of peak velocities and an alteration of the motion pattern of basal slices during diastole, which can be described by a single distinct outward motion, in the LBBB patient a clear alteration of the motion pattern can be observed in all regions of the heart during both systole and diastole. The motion pattern in the LBBB patient starts with a small outward motion of the myocardium in equatorial and basal slices, followed by a motion towards the center of the myocardium. During diastole, the occurrence of a biphasic motion pattern is restricted to the apical and equatorial slices.

**Figure 3:**
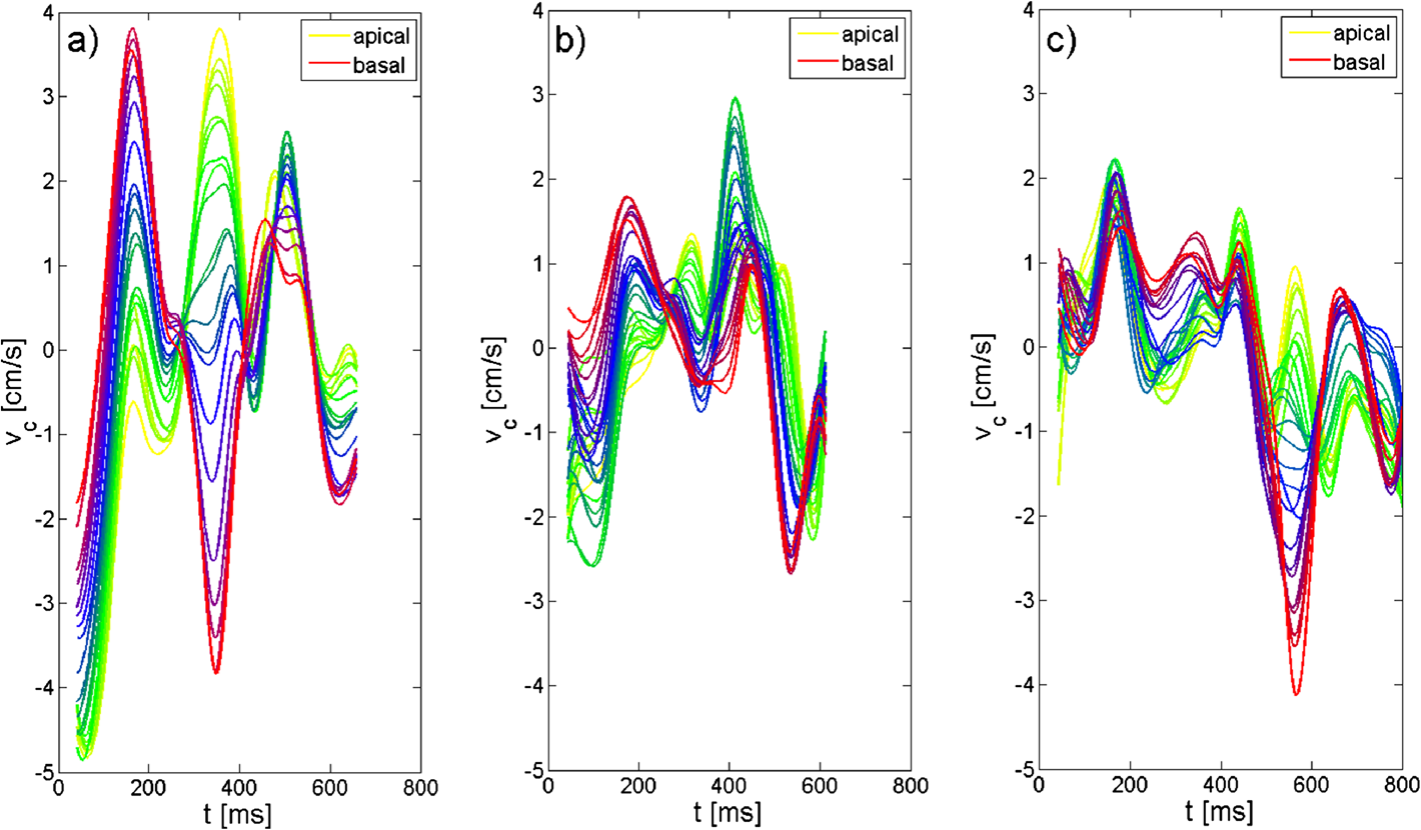
**Circumferential velocity-time curves for a healthy volunteer and both patients obtained by 3D-TPM.** Circumferential velocity-time curves are displayed for all investigated slices for a healthy volunteer (**a**), the DCM patient (**b**) and the LBBB patient. Huge differences between the circumferential motion obtained in the volunteer and in the patients are observed.

Figure 3 displays the circumferential motion exemplary for one volunteer (Figure 3.a), the DCM patient (Figure 3.b) and the LBBB patient (Figure 3.c).

In volunteers, a global motion in counter-clockwise direction occurs at the beginning of systole. Shortly afterwards, the myocardium starts to rotate clockwise in basal and equatorial slices, while it rotates still counter-clockwise in apical slices. This myocardial motion pattern results in the well-known twisting motion of the heart. During diastole, the myocardium starts to rotate clockwise in apical slices, while it moves in counter-clockwise direction in basal slices, thus causing the untwisting motion of the heart. Afterwards, a global motion in first clockwise and then counter-clockwise direction occurs.

As major diffrences between the circumferential motion patterns of volunteers and our two patients reduced velocities during systole and the beginning of diastole can be appreciated. Whereas the DCM patient shows no further alterations in the circumferential v-t curves compared to volunteers, huge differences in the circumferential motion pattern are observed in the LBBB patient. During systole and at the beginning of diastole, the myocardium of both apical and basal regions moves in the same direction thus indicating a loss of twisting motion. In contrast to healthy subjects, during mid-diastole the myocardium moves clockwise in apical slices, whereas it moves counter-clockwise in basal slices.

A summary of the motion quantification parameters calculated from velocity-time curves and torsion rate-time curves can be found in Table 2.

**Table 2:** 3D-TPM velocity based motion quantification parameter provided for the volunteer cohort and for both patients

**Parameter**	**volunteers**		**DCM patient**	**LBBB patient**
	**Mean**	**σ**		
*σ*_*TTP*_^*sys*,1^ [ms]	37.08	22.49	24.02	56.73
*σ*_*TTP*_^*dias*,1^ [ms]	19.52	3.78	6.04	54.10
*σ*_*TTP*_^*sys*,*r*^ [ms]	42.41	8.08	48.69	80.89
*σ*_*TTP*_^*dias*,*r*^ [ms]	37.90	7.44	26.00	51.40
${\overset{―}{ACC}}_{1}$	0.90	0.02	0.77	0.58
${\overset{―}{ACC}}_{r}$	0.71	0.06	0.55	0.40
${\overset{―}{ACC}}_{c}$	0.72	0.04	0.52	0.34
ACC_min,l_	0.32	0.32	−0.17	−0.45
ACC_min,r_	−0.01	0.24	−0.88	−0.31
ACC_min,c_	0.04	0.22	−0.45	−0.66
ACC_max,l_	0.98	0.01	0.97	0.95
ACC_max,r_	0.97	0.01	0.97	0.96
ACC_max,c_	0.97	0.01	0.98	0.94
${\overset{―}{\Delta v}}_{1}$ [cm/s]	13.40	2.30	7.07	4.66
${\overset{―}{\Delta v}}_{r}$ [cm/s]	7.20	0.78	3.42	4.45
${\overset{―}{TUV}}_{1}$	0.86	0.01	0.74	0.74
${\overset{―}{TUV}}_{r}$	0.78	0.02	0.71	0.66
${\overset{―}{TUV}}_{c}$	0.77	0.03	0.69	0.62
peak systolic torsion rate [deg/(cm s)]	10.54	2.78	2.29	−6.27
peak diastolic torsion rate [deg/(cm s)]	−11.85	2.72	1.58	−3.03

### Parameters derived from velocity-time curves

**Figure 4:**
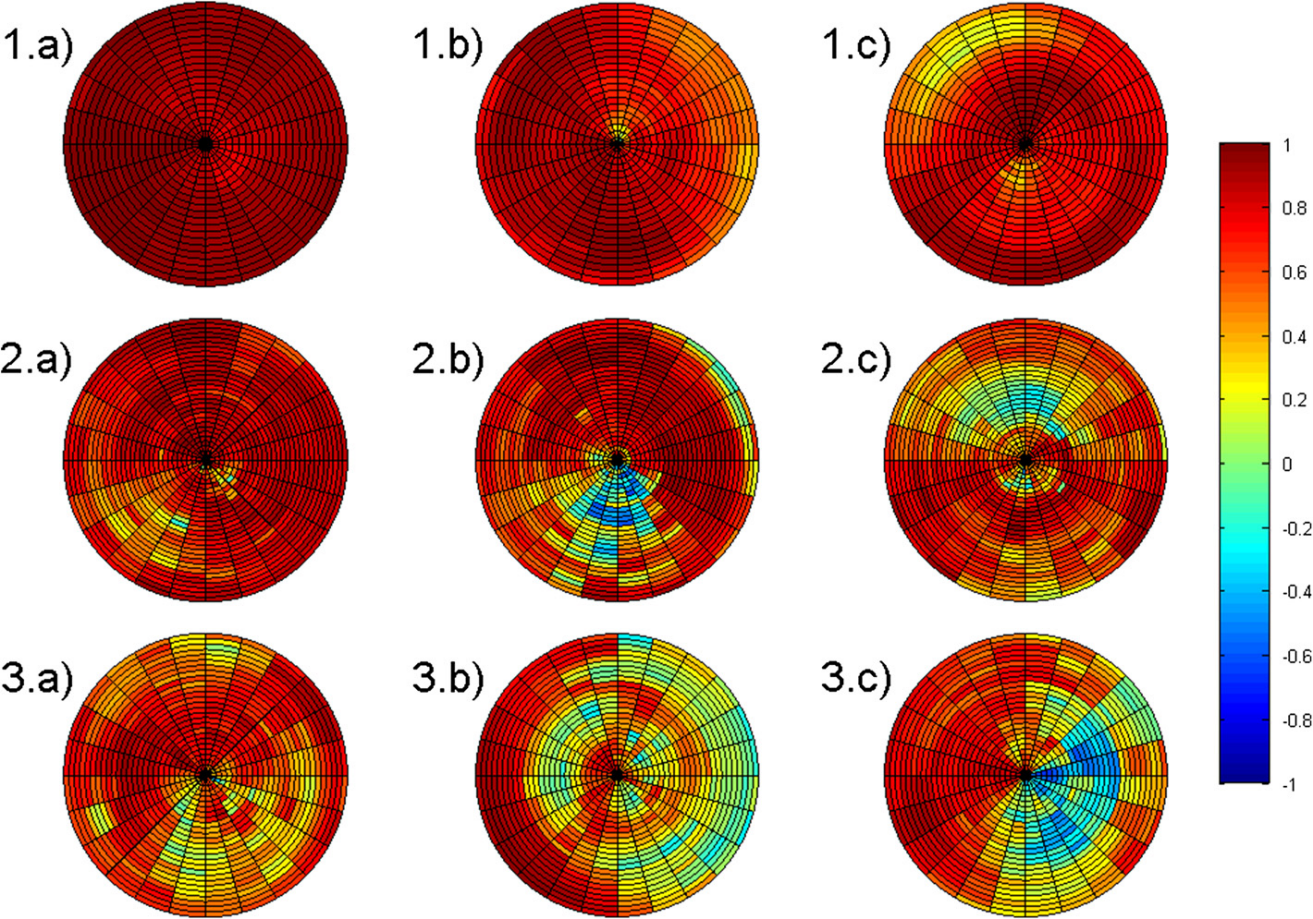
**Longitudinal, radial and circumferential asynchrony correlation coefficients of a volunteer and both patients.** Longitudinal (1.a, 2.a, 3.a), radial (1.b, 2.b, 3.b) and circumferential (1.c, 2.c, 3.c) asynchrony correlation coefficients presented exemplary for one volunteer (1.a, 1.b, 1.c), the DCM patient (2.a, 2.b, 2.c) and the LBBB patient (3.a, 3.b, 3.c). The lateral wall is at the bottom, the inferior wall on the right, the septal wall at the top and the anterior wall on the left.

Low values of σ_TTP_ are obtained in healthy volunteers and the DCM patient, whereas the exemplarily measured LBBB patient shows increased values. The mean and minimum of the asynchrony correlation coefficient is decreased in our two patients compared to the volunteer group for all motion directions. The ACC measured in healthy volunteers reveals synchronous motion in most myocardial segments resulting in mean ACC values higher than 0.7. Figure 4 shows a bullseye plot of the ACC for all investigated segments and slices in a volunteer as well as in our two patients. Whereas in healthy volunteers mostly positive values of the ACC are obtained for all motion directions, the asynchronous segments of the patients show negative values. In most investigated segments and slices, the ACC is higher for the longitudinal motion direction compared to the radial and circumferential motion direction.

The global velocity ranges ${\bar{\Delta v}}_{1}$ and ${\bar{\Delta v}}_{r}$ as well as TUV_l_, TUV_r_ and TUV_c_ are similar in all volunteers, but appear decreased in our two investigated patients.

### Parameter derived from torsion rate-time curves

The torsion rate-time curves shows a relative counterclockwise rotation during systole and a counterclockwise rotation during diastole of the apex against the base, thus revealing positive systolic peak torsion rates ((10.54 ± 2.78) deg/ (cm s)) and negative diastolic peak torsion rates ((−11.85 ± 2.72) deg/ (cm s)) in healthy volunteers. The peak systolic and diastolic torsion rates of the DCM and LBBB patient clearly differ from the volunteer values.

Table 3 presents the motion quantification parameters calculated from the α-t and s-t curves.

**Table 3:** 3D-TPM displacement based motion quantification parameter provided for the volunteers cohort and for both patients

**Parameter**	**volunteers**		**DCM patient**	**LBBB patient**
	**Mean**	**σ**		
BARC	−0.27	0.43	−0.30	0.55
TUS_c_	0.91	0.03	0.71	0.64
TUS_r_	0.86	0.03	0.74	0.79
RVS_max_ [%^2^]	46.37	17.27	44.11	213.18
RVVPS_max_ [%]	65.16	15.54	87.57	220.52
SD(T_onset_) [ms]	14.95	3.24	30.71	61.65
SD(T_peak_)[ms]	49.67	8.61	88.93	89.67
CV [%]	29.39	5.60	74.66	68.95
DiffSLPeakCS [%]	1.36	1.63	2.44	3.58
OS delay SL [ms]	3.22	8.59	−57.45	−59.93
OS delay IA [ms]	2.86	5.30	−80.50	−6.19
OS delay AB [ms]	6.20	14.48	0.00	−29.33
PS delay SL [ms]	46.04	19.85	34.65	−71.12
PS delay IA [ms]	−19.72	17.82	−134.61	15.91
PS delay AB [ms]	−5.37	83.21	111.03	−4.76

### Parameter derived from rotation angle-time curves

In healthy volunteers small or even negative BARC values (BARC ≤ 0.21) are obtained thus showing the twisting motion of the heart. Differences are observed in our two patients. Whereas BARC results in a negative value in the DCM patient (BARC = −0.27), the BARC obtained for the LBBB patient is positive and higher than all BARC values obtained for the volunteers (BARC = 0.55) thus indicating the loss of twisting motion in this patient.

### Parameters derived from strain-time curves

High radial and longitudinal TUS values are observed in the healthy volunteer cohort (TUS_c_ > 0.86 and TUS_r_ > 0.8). The TUS values of our two patients are reduced.

**Figure 5:**
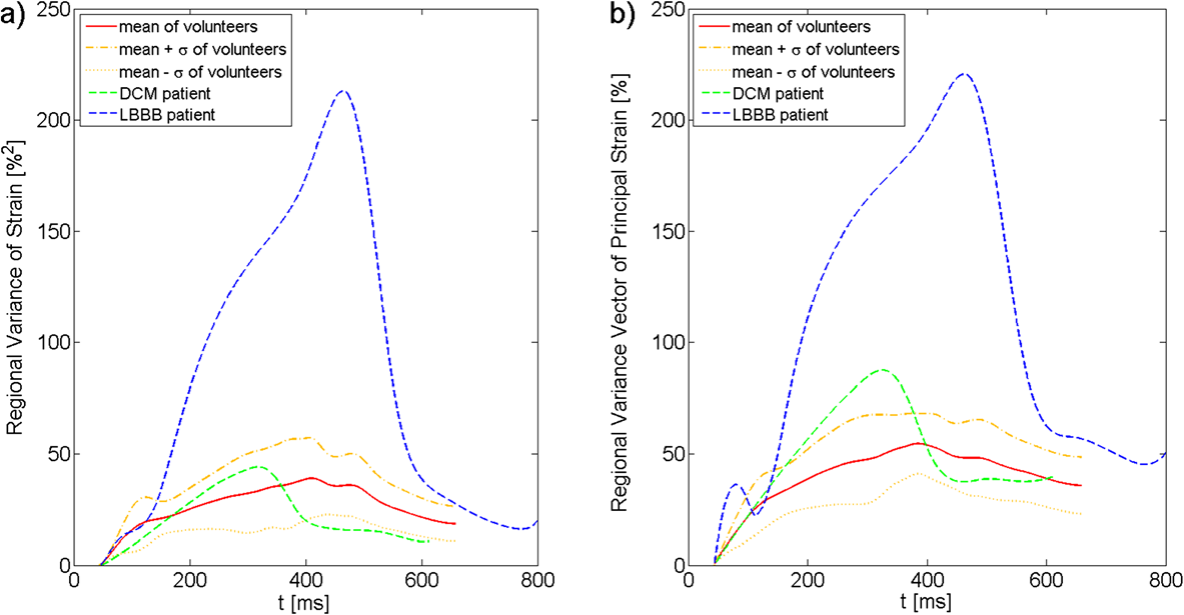
**RVS and RVVPS are displayed exemplary for one volunteer and both patients.** The regional variance of strain (**a**) and regional variance vector of principle strain (**b**) are highly increased in the LBBB patient, whereas the DCM patients shows only small variations from the values obtained in the volunteer.

Figure 5 displays RVS and RVVPS averaged over all volunteers and for both patients. At most time points, increased RVS and RVVPS are obtained in the LBBB patient, whereas the DCM patient shows only slightly increased RVVPS values at end-systole. Increased values in both patients are obtained for RVVPS_max_, whereas RVS_max_ is only increased in the LBBB patient.

The standard deviation of T_onset_ and T_peak_ is increased for our two patients compared to the volunteers thus indicating a higher degree of asynchrony in our two patients.

In our two patients, more than twice as high values are obtained for the coefficient of variation compared to healthy volunteers. The parameter DiffSLpeakCS is only increased in the LBBB patient.

Only the OS delay values in septal-lateral direction are increased in both patients.

The results presented show a trend of increased asynchronicity in our two patients compared to volunteers.

## Discussion

This study shows the feasibility to use 3D-TPM data for the analysis of velocity and displacement based motion quantification parameters. Since all motion quantification parameters are calculated on the same 3D-TPM data, a direct comparison of the performance of the parameters for the detection of motion disorders is possible.

### Parameters derived from velocity-time curves

The values obtained for σ_TTP_ in healthy volunteers are inside the range of standard deviation of σ_TTP_ obtained by Foell et al. [17] for both radial and longitudinal diastolic velocities as well as for the longitudinal systolic velocity (Foell et al.: *σ*_*TTP*_^*dias*,*r*^ = 29.4 ± 8.8; *σ*_*TTP*_^*sys*,1^ = 24.6 ± 21.2; *σ*_*TTP*_^*dias*,1^ = 16.0 ± 5.9 our study: *σ*_*TTP*_^*dias*,*r*^ = 37.9 ± 7.4; *σ*_*TTP*_^*sys*,1^ = 37.1 ± 22.49; *σ*_*TTP*_^*dias*,1^ = 19.5 ± 3.8). In our study *σ*_*TTP*_^*sys*,*r*^ is higher (42.4 ± 8.1) than the value obtained by Foell et al. [17] (22.8 ± 3.7), which can likely be attributed to the increased number of segments used to calculate the standard deviation in our study.

In Foell et al. significant differences between healthy volunteers and patients with DCM or DCM and LBBB have been obtained for *σ*_*TTP*_^*sys*,*r*^, *σ*_*TTP*_^*dias*,*r*^, *σ*_*TTP*_^*dias*,1^. Increased values of these standard deviations are also found in the investigated LBBB patient.

Schneider et al. [18] observed radial ACC values between 0.56 and 1 in healthy volunteers, whereas in our study radial ACC values between −0.59 and 0.98 were observed. Our decreased values in healthy volunteers can likely be explained by the differences of data acquisition, since Schneider et al. have only acquired a single short axis slice, whereas in our study the whole left ventricle is covered, thus including slowly moving slices as well as slices near the basis of the heart, which motion is additionally influenced by the atrial motion pattern. Similar to Schneider et al. [18], the minimum values of the asynchrony correlation coefficients are reduced in our two exemplarily measured patients compared to volunteers. Nevertheless, more patients have to be measured with 3D-TPM in order to reveal statistically significant decreased asynchrony correlation coefficients for patients.

Like in Delfino et al. positive systolic and negative diastolic peak velocities were obtained [19]. The values obtained for the longitudinal and radial velocity ranges are higher in the study of Delfino et al. (Δv_l_= 17.7 cm/s ± 5.2 cm/s; Δv_r_ = 10.3 cm/s ± 3.4 cm/s) than the obtained values in our study (${\bar{\Delta v}}_{1}=13.4\;cm/s\pm 2.3\;cm/s;\;{\bar{\Delta v}}_{r}=7.2\;cm/s\pm 0.8\;cm/s$), whereby ${\bar{\Delta v}}_{1}$ in our study is inside the range of standard deviation from the value observed by Delfino et al. [19]. These differences might by caused, since Delfino et al. used only one short axis acquisition placed at 70% of the distance between apex and basis, whereas in our study the average value over all slices was determined. A trend of decreased velocity ranges in patients was recognized by Delfino et al. [19] as well as in our two patients compared to volunteers.

### Parameter derived from torsion rate-time curves

Like in Petersen et al. a counterclockwise systolic and clockwise diastolic rotation from apex against the basis was observed in healthy volunteers thus resulting in positive systolic and negative diastolic peak torsion rates. Petersen et al. found higher peak systolic ((16.2 ± 4.7) deg/(cm s)) and diastolic ((−15.0 ± 5.7) deg/(cm s)) rotation rates as compared to the values obtained in our study (systole: ((10.5 ± 2.8) deg/(cm s); diastole: ((−11.9 ± 2.7) deg/(cm s)). This might be caused by the different locations of apical and basal slices used by Petersen et al. [21] and in our study. Petersen et al. used the standardized locations according to the 17 segment AHA model [36], whereas in our study the most apical and basal slices of the acquired 3D volume were used.

### Parameter derived from rotation angle-time curves

Like in Ruessel et al. [22] the twisting motion of the heart is apparent in all volunteers. Nevertheless, lower BARC values have been obtained in healthy volunteers in the study of Ruessel et al. [22] (BARC = −0.68 ± 0.22) compared to our study (BARC = −0.27 ± 0.43). These differences may result, since Ruessel et al. have investigated apical and basal slices positioned at one quarter and three quarter of the distance between apex and mitral valve [22], whereas in our study the most apical and basal slice have been used. Which locations along the heart axis provide maximal twisting motion can be further investigated from the 3D data.

Ruessel et al. have additionally shown the feasibility to use the BARC parameter to distinguish between responders and non-responders to CRT [22]. If this result can be confirmed by 3D-TPM needs to be investigated.

### Parameters derived from strain-time curves

TUS_c_ obtained in our study is slightly lower (0.91 ± 0.03) than the obtained value of Bilchick et al. (0.96 ± 0.01) [26]. The decreased value obtained in our study might be caused, since Bilchick et al. have investigated 8–10 contiguous short axis slices, whereas in our study a 3D volume acquisition was used. Both radial as well as circumferential TUS values are decreased in our two patients compared to volunteers. Reduced values of TUS_c_ have also been found in previously described studies investigating 2D short axis views [24,26].

RVS and RVVPS are both highly increased in the LBBB patient when compared to healthy volunteers in this study. Helm et al. has found reduced values of RVS and RVVPS in dogs after biventricular pacing [23] compared to values obtained after left-ventricular pacing and right-atrial asynchronous pacing. Therefore, our study as well as the study of Helm et al. RVS and RVVPS yields smaller values in case of synchronous cardiac motion.

The standard deviation of onset and peak circumferential strain is increased in our two patients compared to healthy volunteers thus indicating an increase of asynchronous motion. Huge differences in T_onset_ and T_peak_ for different segments have also been reported previously by Zwanenburg et al. [27].

Increased values in our two patients compared to healthy volunteers are also obtained for CV, whereas the increase in DiffSLpeakCS appears more pronounced in the LBBB patient. The CV obtained in the study of Nelson et al. is similar to the CV obtained in this study (our study: CV = 29.4% ± 5.6%, Nelson et al.: CV = 28.0% ± 7.1%) [30]. Increased values of CV for patients are obtained in our study and in the study of Nelson et al. [30].

All PS delay vectors of healthy volunteers obtained in our study are inside the range of standard deviation of the study of Zwanenburg et al. [27] (Zwanenburg: PS delay SL = (54 ± 19) ms; PS delay IA = (−17 ± 26) ms; PS delay AB = (2 ± 45) ms; our study: PS delay SL = (46 ± 20) ms; PS delay IA = (−20 ± 18) ms; PS delay AB = (−5 ± 83) ms). No difference to the value obtained by Zwanenburg et al. is also obtained for OS delay AB, whereas OS delay SL and OS delay IA are different (Zwanenburg: OS delay SL = (−12 ± 10) ms; OS delay IA = (−9 ± 9) ms; OS delay AB = (9 ± 7) ms; our study: OS delay SL = (3 ± 9) ms; PS delay IA = (3 ± 5) ms; PS delay AB = (6± 14) ms). These differences might be a result of the higher temporal resolution of 14 ms and the acquisition of only 5 short axis slices used in Zwanenburg et al. [27] compared to a temporal resolution of 37.3 ms and the 3D-TPM acquisition used in this study.

As preliminary study, this study has quantified parameters derived from velocity-time curves, torsion rate-time curves, rotation angle-time curves and strain-time curves from on 3D-TPM data. Advantages expected from the volumetric approach are mainly due to the rather rapid changes of the myocardial motion along the axis of the heart, which may cause huge changes in quantification in case of non-ideal slice positioning. Furthermore, volumetric motion coverage may avoid false-negative patients in cases the motion abnormality is restricted to a location not covered in the multi-slice 2D approach.

One further 3D phase contrast CMR acquisition has been performed by Kvitting et al. [37]. He investigated the velocity of nine predefined points at apical (one point), mid-ventricular (four points) and basal locations (four points). Like our study, the longitudinal velocities were highest at basal slice locations, whereas radial velocities are highest at mid-ventricular slice locations. In contrast to our study, the myocardial motion pattern was not investigated regarding motion quantification parameters, and any rotation angle-time, torsion-time and strain-time curves were calculated. In addition, no respiratory motion compensation was performed by Kvitting et al. due to the long nominal data acquisition times [37].

The long data acquisition time of the applied 3D-TPM sequence (actual measured acquisition time = 34 ± 7 minutes; minimum acquisition time = 25 minutes; maximum acquisition time = 52 minutes) may exceed the maximal scanning time possible in patients suffering from cardiac disease. Furthermore, the long acquisition time may limit the measurement of the motion and other required MR examinations like scar imaging and imaging of coronary veins in a single imaging session. Nevertheless, the information about myocardial scars and the coronary veins is relevant for the determination of a reasonable target position of the lead, therapy-guidance and an assessment of the therapeutic outcome [38-40]. Therefore, further acceleration techniques using a combination of parallel imaging and undersampling in k-t space like k-t SENSE [11] and k-t PCA/SENSE [14] might be investigated in respect to their applicability to reliably quantify myocardial motion in 3D.

In this work, displacements were calculated from velocity data without any model assumptions similar to Pelc et al. [35]. Limitations of this approach are the limited temporal resolution (about 37 ms in this study) and noise amplification. Improved tracking algorithms have been developed including Fourier tracking algorithms and incorporating appropriate local deformation models [41-43]. In future work, the differences between both tracking approaches need to be analysed for the calculation of rotation angle-time curves and strain-time curves.

A further limitation of this 3D volume acquisition might be, that the through-plane field of view was only 6.3 cm in healthy volunteers, 9 cm in the DCM patient and 8.1 cm in the LBBB patient. These through plane field of views cover the whole left ventricle at end-systole. More basal slices were excluded in order to avoid myocardial motion patterns of the atria in some cardiac phases. Future studies might cover a larger field of view or use long axis acquisition geometry for covering the whole left ventricle at end-diastole and evaluate myocardial motion also for the most basal regions of the heart.

Although in this 3D-TPM data acquisition only one navigator at the beginning of the cardiac cycle was used, image quality was still sufficient without respiratory artefacts. Improved respiratory triggering can be performed by e.g. using an additional trailing navigator [44]. This may improve the resulting image quality but further compromise image acquisition time.

Since the number of patients included is very limited, the presented differences between healthy volunteers and patients can only be interpreted as a trend and statistical significance of the observed differences cannot be calculated. In future work, larger patient groups have to be investigated with 3D-TPM to reveal significances between motion quantification parameters derived from healthy volunteers and patients with different cardiac diseases.

## Conclusions

Volumetric tissue phase mapping enables a gapless coverage of the motion characteristics of the myocardium. The resulting velocity data can be applied to derive quantitative motion parameters based on myocardial velocities, torsion, rotation and strain. Similar differences between patients and volunteers are observed as previously reported from 2D-TPM and tagging data. In order to reveal whether 3D-TPM is appropriate to identify myocardial motion differences between healthy volunteers and patients, a larger group of patients has to be investigated.

## Competing interests

VR, JP and AL have a research grant with Philips Healthcare. PE is employed by Philips Healthcare.

## Authors' contributions

AL developed the conception and design of the study, acquired the data, performed the data analysis and interpretation and drafted the manuscript. VR and AB have been involved in the conception of the present study and have made substantial contributions to the interpretation of data and the final approval of the version to be published. JP has been involved in the development of the MATLAB software to determine the motion quantification parameters. Additionally JP has made contributions to the data analysis and interpretation. PE has been involved in the data analysis. GUN, PB and WR participated in the design of the study and interpretation of the data. All authors read and approved the final manuscript.
